# Areas of research to support the system of radiological protection

**DOI:** 10.1007/s00411-021-00947-1

**Published:** 2021-10-17

**Authors:** D. Laurier, W. Rühm, F. Paquet, K. Applegate, D. Cool, C. Clement, the International Commission on Radiological Protection (ICRP)

**Affiliations:** 1 Institut de Radioprotection et de Sûreté Nucléaire (IRSN), Fontenay-aux-Roses, France; 2 Helmholtz Center Munich, German Research Center for Environmental Health, Neuherberg, Germany; 3 Institut de Radioprotection et de Sûreté Nucléaire (IRSN), Cadarache, France; 4 University of Kentucky College of Medicine, Lexington, KY USA; 5 International Commission on Radiological Protection (ICRP) Vice-Chair, Charlotte, NC USA; 6 International Commission on Radiological Protection (ICRP), Ottawa, ON Canada

**Keywords:** Radiological protection, Radiation effects, Radiation dosimetry

## Abstract

This document presents the ICRP's updated vision on “Areas of Research to Support the System of Radiological Protection”, which have been previously published in 2017. It aims to complement the research priorities promoted by other relevant international organisations, with the specificity of placing them in the perspective of the evolution of the System of Radiological Protection. This document contributes to the process launched by ICRP to review and revise the System of Radiological Protection that will update the 2007 General Recommendations in ICRP Publication 103.

## Introduction

Since 2011, the International Commission on Radiological Protection (ICRP) has included in its strategic plan a priority to “identify and encourage the research needed to support radiological protection.” This priority was reiterated when the strategic plans were revised in the “ICRP Strategic Priorities 2020–2024” and published online in 2020 (ICRP 2020b).

This document presents the ICRP's vision on “Areas of Research to Support the System of Radiological Protection”. It updates the previous version published in 2017 (ICRP 2017c). Research needs are grouped into three main areas: research to support radiation risk assessment; research to support dosimetry; and, research to support the application/implementation of the System of Radiological Protection. In each area, a distinction is made between research needed in the short/mid-term (in support of ICRP’s next General Recommendations) and in the longer term (beyond 10 years).

## Research to support radiation risk assessment

### Short/mid-term research

#### Classification of radiation health effects

The effects of ionising radiation on human health are currently classified into two broad categories, referred to as “tissue reactions” and “stochastic effects” as described in ICRP *Publication 103* (ICRP 2007). The objective of the System of Radiological Protection is to prevent tissue reactions and to limit the risk of stochastic effects to the extent reasonably achievable. However, with the evolution of knowledge on radiation-induced health effects, this simple classification may require reconsideration based on the most recent results of scientific research.

#### Better characterisation of tissue reactions

Tissue reactions are due to an injury in populations of normal cells after radiation exposure. They are characterised by a threshold dose and an increase in the severity of the reaction as the dose is increased further. In 2012, ICRP has provided a review on tissue reactions in *Publication 118* (ICRP 2012). Currently, tissue reactions include a wide variety of diseases, some specific to radiation occurring in the short term after high-dose exposure, and others being non-specific (such as diseases of the circulatory system) and observed several years or decades after exposure. The experience gained in radiotherapy and interventional procedures on the consequences of radiation exposure on tissues could be better consolidated. Further research is needed to have a better understanding of tissue responses, their variation between individuals, and the relationships between dose and the probability of occurrence or the severity of effects, which form the foundation for the determination of threshold doses.

#### Stochastic effects and radiation detriment

Stochastic effects of ionising radiation include cancer and heritable effects. Radiation detriment is a concept used to quantify the harmful stochastic effects of low-level radiation exposure to the human population. It aims to integrate all stochastic effects, considering the probability of occurrence of a disease after radiation exposure, and taking into account its severity in terms of lethality, quality of life, and years of life lost. The concept of radiation detriment was introduced by ICRP in *Publication 60* (ICRP 1991) and has been confirmed in *Publication 103* (ICRP 2007). During the last 20 years, advancements in science and technology have led to new results and insights that warrant an update of detriment, and highlight the need for continued research, especially in support of the following points:

- Cancer risk models and tissue weighting factors

There is growing evidence from epidemiologic studies of dose–risk relationships at dose levels down to about 100 mGy or less, for all cancers and for several specific cancer sites (see for example Grant et al. 2017; Richardson et al. 2015; Little et al. 2017; Lubin et al. 2017; Hauptmann et al. 2020; Wakeford and Bithell 2021). Although risk models are available for many cancer sites that incorporate modifying factors such as sex, age at exposure and time since exposure (Cahoon et al. 2017; Brenner et al. 2018, 2020; Mabuchi et al. 2021), there are still large uncertainties related to radiation-induced risks at low doses and the shape of the dose-risk relationships. Also, the transfer of risk estimates between different populations is still uncertain. Further epidemiological studies with extended follow-up can provide new insight on these issues and should help in consolidating the assessment of cancer risks associated with low doses, with an improved characterisation of modifying factors of the dose-risk relationships. Also, these results should improve the quantification of the relative contribution of cancer sites to the overall radiation detriment, and therefore, should improve the basis for setting tissue weighting factors, *w*_T_.

- Dose-rate effects and cancer

Calculation of radiation detriment is largely based on cancer risks obtained from the Japanese atomic bomb survivors who were exposed to ionising radiation at high dose-rate. In contrast, except in the medical field, radiological protection is generally concerned with exposure situations involving lower doses and dose rates, even if cumulated doses over 100 mGy may be encountered in specific situations. The question of whether low dose-rate exposures are less carcinogenic than high dose-rate exposures, given the same dose, remains controversial (Rühm et al. 2016; Shore et al. 2017; Leuraud et al. 2021). Therefore, research on the dose-rate dependence of cancer risk continues to be important, with a focus on epidemiological studies among human cohorts, backed by radiobiological studies on the mechanisms of cancer development. Effects of FLASH radiation therapy using ultra-high dose-rate exposures would also deserve further research (Griffin et al. 2020).

- Impact of non-radiation factors in detriment calculations

Besides lifetime cancer risk for various tissues and organs, calculation of radiation detriment includes several additional factors not related to radiation exposure, such as lethality, quality of life, and years of life lost (ICRP 2007; Cléro et al. 2019). Since these factors depend on the changes in lifestyle, advances in diagnostic technologies and cancer treatment, and status of health care system, they require periodical update (Breckow et al. 2018; Breckow, 2020). Alternative approaches to calculating radiation-induced detriment and to adjusting for the severity should be explored and need further research (Shimada and Kai 2015).

- Potential impact of diseases of the circulatory system on radiation detriment

Some non-cancer effects might be better classified as stochastic effects. Evidence for increased risk of diseases of the circulatory system following exposure to ionising radiation at low to moderate doses and dose-rates has accumulated from epidemiological studies over the recent decade (Azizova et al. 2015; Tapio et al. 2021; Little et al. 2021). These results show an increase in the probability of occurrence with dose, with no variation in severity. Nevertheless, certain aspects of the epidemiological evidence require clarification, the biological mechanisms are still unclear, and risks at low doses are associated with large uncertainties. Long-term surveillance of paediatric cancer survivors who underwent radiation therapy may provide some informative data. Further research should provide the basis for discussing the pertinence and feasibility of including the diseases of the circulatory system in radiation detriment, together with elements necessary for quantifying their risks.

- Effects of radiation from in utero exposure

The issue of health effects of in utero radiation exposure are especially important for the medical profession. Much of the current guidance relies on animal research and limited epidemiological data (ICRP 2003a), but some new results were published in the recent years. Results show smaller head and chest size at birth, increased risks of paediatric leukaemia and cancer, increased systolic blood pressure at adolescence, and increased risk of cancer in women at late adulthood (Nakashima et al. 2007; Hatch et al. 2017; Sugiyama et al. 2021; Wakeford and Bithell 2021). Further research is needed in understanding the long-term health effects from in utero low dose exposures.

- Heritable effects of radiation on offspring and next generations

The issue of potential effects for the offspring and subsequent generations is a recurrent major concern for the general public and a particular one for parents (and potential future parents) exposed to ionising radiation from occupational, medical, or environmental sources. Today, there is little evidence from epidemiological studies to suggest the existence of heritable deleterious effects resulting from radiation exposure in humans (Yeager et al. 2021; Yamada et al. 2021). However, heritable risks are included in overall stochastic risks based on evidence in experimental animals (UNSCEAR 2001). There is still a lack of knowledge about the fundamental mechanisms for potential radiation-induced genetic diseases, particularly multifactorial diseases that largely manifest later in life, and about the contribution of epigenetic processes in adverse outcomes, if any. Further research is needed in genetics, epigenetics, radiobiology, toxicology, and epidemiology, to better characterise and quantify potential heritable effects among humans and non-human species.

- Uncertainty analysis

Estimates of radiation-induced risks at low to medium dose levels are associated with large uncertainties. Sources of uncertainty can be identified (e.g., study design, selected population, exposure assessment, dosimetric assessment, health outcome assessment, confounding factors, statistical, and modelling methods) but their combined impact on risk estimates is rarely quantified (UNSCEAR 2015, 2020; Zhang et al. 2020). The process of quantifying radiation detriment does not currently consider the underlying uncertainties. Methodologies to estimate health risks from exposure to ionising radiation incorporating associated uncertainty should be further developed, as well as approaches to propagate these uncertainties in risk estimates used as underlying assumptions of the System of Radiological Protection.

#### Individual response of humans to radiation

Over the last two decades, there has been progressively more consideration given to various factors contributing to differences in sensitivity of population groups to ionising radiation exposure (Applegate et al. 2020). For example, sex, age at exposure, and attained age can be significant modifiers of the relationship between dose and cancer or non-cancer risks (Grant et al. 2017). Differences due to some lifestyle characteristics, such as smoking, have also been observed (Cahoon et al. 2017), which raises the general question of how ionising radiation interacts with other risk factors. This question is fundamental to the transfer of risks between populations with different background cancer rates. Increasingly, the paediatric oncology research community has identified cancer predisposition syndromes in children, mapped out recommended management for these children and their families, and suggested avoidance of ionising radiation in a growing number of these exposure situations (Brodeur et al. 2017). Some risk factors may be transient due to hormonal, medication, or other factors that increase tissue radiation sensitivity. The role of genetic and epigenetic differences in determining individual sensitivity also has the potential to significantly influence radiological protection and, thus, requires further research. Related ethical aspects also deserve dedicated research.

#### Radiation effects on non-human biota

The focus of protection for non-human biota in the natural environment is on population viability, including gross impairment of reproductive capacity and effects on future generations. Concepts for the protection of non-human biota against radiation-induced effects have been developed by ICRP, as documented in a series of publications (ICRP 2008, 2021b; ICRP 2017a). Further research is needed to relate exposures, doses, and effects on population viability for non-human biota, accounting for differences in sensitivity between organisms and life stages (Garnier-Laplace et al. 2015). Over the longer term, additional knowledge may also be required on the effects of ionising radiation on the structure and function of ecosystems, which may lead to a more holistic consideration of both biodiversity and ecosystem services.

### Long-term research

#### Basic research

There is a need for further studies on the mechanisms of low dose effects at the molecular, cellular, and tissue levels along with the development of dose–response models that take these mechanisms into account. Biological samples of normal and diseased tissues taken during epidemiological and experimental studies have the potential to link changes at tissue, cellular, and sub-cellular levels to observed health effects (Hall et al. 2017). Research related to the identification of radiation signature(s) for specific cancers should be continued, as well as that dedicated to the identification of biomarkers or the characterisation of non-targeted mechanisms. These data, combined with multilevel analytic approaches such as systems biology, should improve our understanding of radiation effects. Integration of approaches, such as considering Adverse Outcome Pathways (AOP, an approach that identifies the sequence of events required to produce a toxic effect when an organism is exposed to a substance) (Preston et al. 2021; Chauhan et al. 2021), should help in identifying the causal sequence between exposure to ionising radiation and disease. Basic research is also essential to improve fundamental knowledge and maintain competences in the field of radiological protection.

#### Effects of combined exposures

Situations involving exposure to ionising radiation are rarely isolated, and the potential impact of other pollutants on the risks associated with radiation is difficult to assess. There is a clear need to consider radiation in the context of our overall environment. An example is to better quantify the combined effect of exposure to radiation and smoking (Furukawa et al. 2010). Approaches to consider the exposome should be developed in the radiation field, to better allow consideration of potential interactions with other factors, or cocktail effects (Wild 2012). This research requires a rapprochement of expertise in toxicology between the radiological and chemical fields. Also, the impact of combined exposure to ionising radiations of different qualities requires more investigation.

## Research to support dosimetry

### Short/mid-term research

#### Relative biological effectiveness, quality factor and radiation weighting

In the current System of Radiological Protection, Relative Biological Effectiveness (RBE) is expressed in terms of the derived quantities such as the Quality factor, Q, and the radiation weighting factor, *w*_R_ (ICRP 2003b). RBE is experimentally determined by type of ionising radiation, density of energy deposition, dose, dose rate, fraction pattern of the exposure, and biological endpoint (Ujeno 1983; Rossi and Zaider 1990; Edwards 1999). RBE is also influenced by the reference radiation and the dose delivered in the experiment. An expansion of data to include not only genetic alteration but also tissue responses in endpoints is encouraged. In addition, specific attention should be given to low-energy electrons such as soft beta particles, electrons produced by low-energy photons, and Auger electrons which have very low penetrability in biological tissues (Paquet et al. 2013). Incorporation of radionuclides emitting such low-energy electrons into a cell may induce a heterogeneous deposition of energy within the cells that is effective in inducing cell killing or cell mutation. Currently available RBE data need to be extended to cover the range of electron energies.

RBE data for non-human biota have been derived for the specific cases of alpha-particle-emitting radionuclides and tritium beta particles, and for the endpoints of relevance to animals and plants (ICRP 2021b). Taking into account that RBE data used for humans are mainly derived from animal studies, it would be worthwhile to extend these data and to adopt a general approach that would allow the derivation of radiation weighting factors for both humans and non-human biota, considering different endpoints (stochastic effects, tissue reactions, and effects for biota populations and biodiversity).

#### Appropriate dosimetric quantities for medicine and other applications

Effective dose (*E*) was developed by ICRP as a risk-adjusted dosimetric quantity for the management of protection against stochastic effects, principally cancer, enabling comparison of estimated doses from all types of ionising radiation with dose limits, dose constraints, and reference levels (ICRP 2007). In medicine and other applications, *E* may not be applicable especially when the procedure is specific for a restricted group of patients which differ largely from the reference person in terms of age, sex, size, and metabolic functions (important with regard to biokinetics of radiopharmaceuticals in nuclear medicine). This can be the case for patients with impaired organ function or for patients with organ ablation (e.g. thyroid), as well as for some examinations conducted only for patients of a specific sex (like mammography, or diagnosis of prostate cancer). In all these cases, the averaging of male and female organ doses, and using the reference person anatomy, metabolism, and weighting factors which have been averaged over the whole population, limits the use of *E* in discussions with individual patients and their clinical referrers. A dose quantity similar to *E* could instead be specified separately for males and females of different ages, taking account of differences in radiation detriment with age at exposure, and allowing consideration of differences from reference body sizes (ICRP 2021a). Research and consideration of the best formulation of *E* for use in patient dose assessment and for other situations of exposure would help determine the most appropriate future changes to this risk-related quantity.

#### Dosimetry in emergency situations

The current ICRP dosimetry system focuses on regulatory compliance and optimisation of protection at low doses using effective dose to assess exposures. There is a need to expand the current system for performing radiological assessment in emergency situations. In such situations, the focus will be on individual prospective and retrospective dose assessment as well as assessments for population groups. The requirement is to define approaches that consider both stochastic effects and tissue reactions, situation-specific conditions such as thyroid blocking or contaminated wounds, and individual characteristics (such as iodine deficient diet in affected region for example). Complexities include the setting of exposure levels that will appropriately avoid severe tissue reactions, considering acute doses and protracted external and internal doses. There is also a need to consider appropriate target tissues and/or regions within tissues in relation to tissue reactions (see ‘Definition of dosimetric targets in organs or tissues, below). More research is required to develop appropriate approaches and systems of response.

## Long-term research

### Definition of dosimetric targets in organs and tissues

The averaging of absorbed doses in different tissues or organs of the human body is the basis for the definition of the protection quantities which are used for limiting stochastic effects at low doses (ICRP 2007). The extent to which the mean absorbed dose is representative of the local absorbed dose depends on several factors, including the penetrability of the incident radiation in the body, the structure of the organs and the distribution of radioactive sources and the sensitive cells. For ionising radiation with low penetration or limited range (low-energy photons, charged particles), energy deposition in biological tissues can be very heterogeneous (Paquet et al. 2013) and therefore target cells responsible for the induction of a damage need to be identified.

For stochastic effects, the target cells are defined as homogeneously distributed in most of the organs, except in the cases of the human respiratory tract, the alimentary tract, the urinary bladder, the skeleton and the skin, for which doses are calculated to specific cells layers. The need to supplement this list with other organs with complex internal structures (e.g. inner medulla and surrounding cortex in kidneys, adrenal glands, testes, prostate; grey and white matter in the brain) may be investigated, including for applications in nuclear medicine. Also, research may be needed to revisit the notion of “target” to better reflect the possible contribution of non-targeted mechanisms in the development of cancer.

For some tissue reactions, the location of target cells is not yet defined for dosimetric purposes (Gössner 2003). Dosimetric targets need to be better identified and specified in the phantoms being developed, considering tissue, sex and age dependence. For diseases involving multiple organs, such as diseases of the circulatory system, consideration of multiple targets may be investigated, based on the evolution of knowledge on the biological mechanisms.

### Strengthening dosimetric targets and methodology for the protection of the environment

Radiological protection of the environment deals with detrimental effects of ionising radiation exposure traceable at population levels, which can be explicitly seen soon after the exposure. Biological endpoints considered in environmental radiological protection are focussed on immediate or early effects in the exposed organisms or ecosystems. The goal of radiological protection in the environment is to limit the radiation impact on biological variety, ensuring conservation of species, health and status of natural habitats, communities and ecosystems (ICRP 2008, 2014a). Currently, protection of the environment is addressed by use of a restricted set of Reference Animals and Plants (RAP) in combination with Derived Consideration Reference Levels (DCRL), representing ranges of dose rates to biota associated with harmful effects of ionising radiation exposure, assuming stationary conditions. Dose rates are calculated as averages for whole organisms, using highly simplified body shapes described as spheres and ellipsoids, and the simplest of biokinetic considerations using equilibrium concentration factors (ICRP 2008, 2009, 2017a).

Due to the wide diversity of living organisms, the definition of targets within tissues and the associated dosimetric modelling could not be considered as a reasonable goal for all species. However, for certain biological endpoints, like reduced fertility or fecundity, or for exposures resulting in highly inhomogeneous dose distributions in the body, the use of averaged whole-body dose may be insufficient or non-informative, thus requiring assessment of doses in specific organs or tissues for organisms of interest under realistic exposure conditions and accounting for realistic behaviour of the organism. Establishing radiological protection criteria for such situations may require further development of more realistic dosimetric and biokinetic models, at least for larger animals, extension of the set of RAPs and of exposure situations required for effective radiological protection of the environment.

Research is required to improve dose assessments for non-human biota, considering the transfer of radionuclides through the environment, delineation of the external radiation field and exposed group, and radionuclide concentration ratios between organisms and the environment. Consideration of uncertainties in dose assessment will help focus attention on the most important factors.

More detailed dosimetry is likely to be necessary when considering animals as veterinary patients. While not always warranting the sophistication of the anatomical models used in human dosimetry, some simplified versions of such models could be developed and refined as necessary.

### Biokinetic models of radionuclides and radioactive substances in human tissues

Biokinetic models describe the time-dependent absorption, distribution, and retention of radionuclides in the body. Biokinetic data are used to calculate the dose to organs and tissues and then to calculate the effective dose. ICRP has developed over the years a series of models describing the deposition and absorption of radionuclides in the human alimentary tract and the human respiratory tract and their distribution and retention in the body (ICRP 1994, 2006, 2015b, 2016b, 2017b, 2019).

The last generation of models produced by ICRP considers the recycling of the elements and are more physiologically realistic than before. These models also define age-dependant biokinetic parameters, to allow the calculation of effective dose to workers and members of the public. In the medical field, improved dynamic models will facilitate the estimation of doses from new radiopharmaceutical products.

A new set of data remains to be produced for the description of the transfer of radionuclides to the fetus from the mother, and to the new-born, infant and toddler through maternal milk. Related models were produced about 20 years ago using the best available knowledge (ICRP 2001, 2004, 2015a). They need to be completed by new data describing more precisely these processes.

## Research to support the application/implementation of the System of Radiological Protection

### Both short/mid and long-term research

#### Development and use of radiation technologies

The use of radiation is constantly expanding and changing, and this poses challenges for radiological protection and the delivery of medical and veterinary care. Thus, research and development in the various uses of ionising radiation and radioactive material, and its alternatives, is crucial to appropriately establish an effective System of Radiological Protection for all domains, including medical, veterinary, industrial, and academic activities. Implementation science is the study of methods to promote the adoption and integration of evidence-based practices, interventions, and policies into routine health care and public health settings.

- Medical use implications in treatment and protection

Research and collation of information is continuously required in relation to the best use of ionising radiation and radioactive materials in medical diagnosis and treatment. The medical field includes a large diversity of uses including dental radiology, FLASH and other new beam delivery modalities in radiation therapy, nuclear medicine, and interventional radiology, and the rapid development of new techniques, such as vectorised radiotherapy and theragnostic approaches. They involve very different dosimetry approaches, levels of dose and dose-rate, and heterogeneity in dose distribution in the body. Of particular importance is the assessment of exposures and implications for vulnerable population groups, including the understanding of individual responses to radiation exposure which may impact protection and treatment protocols. Specific attention should be given to patients with cumulative imaging examinations or repeated fluoroscopic-guided interventional procedures reaching doses above 100 mSv (Rehani et al. 2019), with considerations of appropriate time intervals between examinations. Development of dose registries of both diagnostic imaging and radiotherapy patients available to the epidemiology research community should be considered to support further research. Also, research is required to better relate possible detrimental effects of ionising radiation exposure to the clinical benefits of diagnosis or therapy.

- Veterinary practice implications in treatment and protection

Individual animals should be protected in veterinary applications of ionising radiation. The area of veterinary practice is rapidly expanding, with many radiological medical procedures originally developed for human use being employed for animals (Pentreath et al. 2020; IAEA 2021; ICRP 2020a). This raises questions regarding how and why ionising radiation is used in veterinary practice (e.g., in situations that may not be medically indicated). Research is needed to inform issues in justification (appropriateness criteria, characterisation of outcomes, and cost-effectiveness analyses for both imaging and radiation therapy), optimisation (imaging protocols and diagnostic reference levels for imaging and radiotherapy modalities for veterinary animals) and dose limitation, taking into account the radiological protection of the animal owners and handlers who are exposed while providing care, the workforce, type of procedure, and potential for environmental contamination with use of radiopharmaceuticals.

- Industrial and academic applications, including Naturally Occurring Radioactive Material (NORM)

The use of ionising radiation and radioactive materials is constantly expanding, and new developments, such as reuse and recycle of NORM, can have implications for radiological protection. Further investigations are needed to better characterise exposures from such new sources of exposure to radiation and investigate any radiological protection implications in situations where there may be relatively low awareness of radiological protection principles.

- Natural sources of radiation exposure

Individuals can be exposed to natural sources of ionising radiation in a wide variety of situations and circumstances (UNSCEAR 2010), some of which may be new or novel radiation environments. Residential radon often represents the main component of the public dose and should be considered in the more global context of indoor air quality. Changes to buildings, including for example ventilation and construction materials, can significantly alter radon exposures (ICRP 2014b). Other examples include air travel (ICRP, 2016a) and the potential expansion of space exploration and space tourism (ICRP 2013). Further research is needed to characterise the doses and risks specifically associated with these exposure situations, and to support an understanding of these situations on specific groups of population and the protection implications, including issues related to tolerability and reasonableness of risk.

#### Ecosystem protection

ICRP has taken substantial steps towards protection of the environment over the last 15 years (ICRP 2008, 2009, 2014a, 2021b). This topical area, in conjunction with research on radiation effects and developments in dosimetry, is designed to support continued development and consideration of an improved and more holistic approach to ecosystem protection. This includes the natural environments and “manufactured” environments where there is human management such as farms, parks, and even larger areas that have substantial human oversight. Broadly, this includes plants and animals, as well as ongoing developments in environmental protection such as sustainability of natural resources, and the impact that uses of ionising radiation may have on the ecosystem both in the short and long term. This research should help to better inform the implementation of environmental radiological protection principles. Especially, there is a need to better understand the pathways, transport and transfers of radioactive materials from a source to a receptor, supporting complementary research on dosimetry and effects (at all organisational levels–from individual to community), and their management in the environment for non-human biota. Research is needed to provide an understanding of the impact of ionising radiation exposure on biodiversity and on ecosystem services, including natural resources across a broad range of natural environments as well as environments impacted and shaped by human activities.

#### Research needs for the application of the system of radiological protection

The system of protection is grounded on science, ethics, and application (ICRP 2018). This topical area broadly covers the many types of social, communication, and technology assessment research that contribute to the successful application and communication of the System of Radiological Protection. There must be information to support the System for innovation, validation, testing and simulation of new technology and software, and new applications of older technology that require research and quality management programmes.

- Implications of Artificial Intelligence (AI) to radiological protection practice

Developments in application and use of AI, notably machine learning, are rapidly expanding in many areas, especially in medical applications (Geis et al. 2019). Numerous applications are already under development for selection (or deselection) of patients, automated treatment planning, and optimisation of imaging protocols. The later has already shown improvement in standardisation and optimisation when compared to expert radiologists (Mukherjee et al. 2020; Pinto et al. 2021). These developments should lead to improvements in the accuracy of doses, prediction science and in the quality of medical decisions. However, specific local and national research and validation, training, policy, and ethical oversight have yet to be in place (Larson et al. 2021). The complexity of the medical work environment, the need for teamwork, and integration of multiple user interfaces for equipment and software, require a new and sophisticated implementation and quality assurance process (Levenson 2012; Li et al. 2020). Beyond medical care, creation of digital twin technologies is being considered for monitoring of workers, control of systems containing radioactive material, and activation of safety systems. While the impact of these developments may be difficult to predict in advance, it may have significant implications for radiological protection of patients, occupationally exposed individuals, and the public. Research and reviews, including ongoing ethical reviews, are needed to better understand the development and implementation of AI technologies, and how these may need to be reflected in the System of Radiological Protection.

- Social science research on perception and understanding of radiation and its use

There are many lessons to be learned from public perceptions and behavioural responses to radiation emergencies and contaminated environments resulting from human activities, as well as information from non-radiological events such as natural disasters and the SARS-CoV-2 pandemic.

Situations where radiological risks are to be addressed are generally complex, multi-disciplinary, and multi-dimensional, and need to be managed in the context of other risks (e.g., chemical, biological, economic, societal). Additionally, the implementation of protective actions (for patients, workers, the public, and the environment) can result in additional risks affecting human health, due to changes in lifestyle for example. Therefore, radiological risk must be balanced against other risks, when deciding on the best protection strategy under the prevailing circumstances. Because the risks to be considered and balanced are so diverse, research is needed on developing robust and validated approaches for assessing diverse risks in a common framework. This would help in promoting an integrated and properly graded all-hazards approach in the justification and optimisation of protection. For example, insights into the role played by economic interests, sustainable development, and well-being could be investigated to identify their implications for the System of Radiological Protection.

- Mechanisms for stakeholder involvement and communication science

Radiological protection is largely dependent on shared decision-making with the relevant stakeholders. Such decision-making relies upon trust, as exemplified in everyday practice in healthcare (Elish and Watkins 2020). Although decades of research have been devoted to perception, risk, trust, decision-making, cultural competencies, and on ethical values in medicine and other social science domains, continued work is needed when applied to the understanding of radiation and its use (Amoore 2020; Siegrist 2021). Engagement involves providing timely, accurate, and appropriate data and information that is understandable for the stakeholder group. It also involves discussions of uncertainties, disclosure of accidents, and listening to concerns (Goske et al. 2012). Long-term recovery from the Fukushima Daiichi accident has demonstrated the importance of establishing and maintaining dialogue with stakeholders and facilitating the co-expertise process between authorities, experts and stakeholders in creating viable and sustainable outcomes (ICRP 2020c). Continuing efforts are needed to further understand and apply strategies for stakeholder engagement, risk communication, and building competence and resilience in groups and communities, tailored to the various exposure situations (Clement et al. 2021).

- Ethics

Foundational to radiological protection are underlying ethical principles, including the core and procedural values given in *ICRP Publication 138* (ICRP 2018). Within this broad area are the particular issues for dealing with specific individuals, research subjects, populations, medical and veterinary practitioners and patients, and non-human biota in the environment. There is less evident distinction between clinical research and practice today requiring more care in understanding and defining consent and shared-decision-making (Elish and Watkins 2020). Psychological consequences of exposure to ionising radiation should also be considered. In addition, as research better quantifies individual-level risks to radiation from both environmental and genetic factors, the System of Radiological Protection will face new ethical challenges (Applegate et al. 2020). Further research and guidance on these ethical aspects is needed to strengthen the System of Radiological Protection.

- Behavioural science

Radiological protection depends upon individual, organisational, and societal (cultural) actions, attitudes, and behaviours, including economics and response to stressful or unknown situations. Further developments are needed, for example, in applications and understanding of the complementary discussions of a graded approach to protection, responses and reactions to unanticipated and emergency situations, the development and maintenance of a radiological protection culture, and optimisation of protection where all societal and environmental factors are appropriately balanced.

## Conclusion

ICRP has embarked on a review and revision of the System of Radiological Protection that will update the 2007 General Recommendations in ICRP *Publication 103* (Clement et al. 2021). This is the beginning of a process that will take several years, involving open and transparent engagement with organisations and individuals around the world. While the System is robust and has performed well, it must adapt to address changes in science and society to remain fit for purpose.

The present article is meant to both complement and build on the more descriptive recent publication which identifies areas for engagement, discussion, and review of the System of Radiological Protection, with the radiation protection community (Clement et al. 2021). It presents ICRP's vision of “Areas of Research to Support the System of Radiological Protection”. In doing so, the ICRP aims to harmonise and highlight research priorities that are promoted by other relevant international organisations (Kreuzer et al. 2018; Muikku et al. 2018; Hoeschen 2018; Schneider et al. 2018; Bouffler et al. 2019; NERIS, 2019; Perko et al., 2019; Harrison et al. 2021; Impens and Salomaa 2021), with the specificity of putting them in the perspective of the evolution of the System of Radiological Protection. The research needs identified cover a wide range of disciplines, some overlapping, including but not limited to artificial intelligence, communication science, dosimetry, ecology, epidemiology, ethics, medical imaging and radiotherapy, modelling, radiobiology, social sciences, technology development, toxicology and uncertainty analysis. It distinguishes between research needed in the short/mid-term (in support of the next General Recommendations) and in the longer term (beyond 10 years). Beyond this distinction, the Commission is conscious that the anticipation of research yielding results in the short-term does not preclude continuation over the long-term.
